# *CTLA-4* +49 A/G Polymorphism and the Risk of Lung Cancer: a *Meta*-analysis

**DOI:** 10.3779/j.issn.1009-3419.2021.102.09

**Published:** 2021-03-20

**Authors:** Zhengliang WEI, Shaoqin ZHANG, Jian HU

**Affiliations:** 1 Department of Cardiothoracic Surgery, Shengzhou People's Hospital (the First Affiliated Hospital of Zhejiang University Shengzhou Branch), Shengzhou 312400, China; 2 Department of Cardiothoracic Surgery, The First Affiliated Hospital, Zhejiang University, Hangzhou 310006, China

**Keywords:** CTLA-4, Polymorphism, Lung neoplasms, *Meta*-analysis

## Abstract

**Background and objective:**

Lung cancer is one of the malignant tumors. Gene mutations associated with cellular immune function and regulating the activation and proliferation of immune cells. Several publications have explored the relationship between cytotoxic T lymphocyte antigen-4 (*CTLA-4*) +49 adenine (A)/guanine (G) polymorphism and susceptibility of lung cancer, but the results remain controversial. Thus, we performed this *meta*-analysis to derive a more comprehensive estimation of the relationship.

**Methods:**

All articles addressed lung cancer and polymorphisms of *CTLA-4* were searched from the PubMed, EMBASE databases published up to June 29, 2019. Odds ratios (ORs) with 95% confidence intervals (CIs) were used to assess the strength of association. Publication bias of relevant studies was examined via *Begg's* test and funnel plots.

**Results:**

The *meta*-analysis included 8 case-control studies covering 4, 430 lung cancer patients and 5, 198 healthy controls from September 2008 to April 2020. The overall eligible data indicated that *CTLA-4* +49A/G polymorphisms did not correlate with the elevated lung cancer risk in all genetic comparison models (dominant model: OR=1.037, 95%CI: 0.925-1.161; recessive model: OR=0.968, 95%CI: 0.888-1.055; allele model: OR=0.992, 95%CI: 0.933-1.054; homozygous model: OR=0.980, 95%CI: 0.857-1.121; heterozygous model: OR=1.023, 95%CI: 0.906-1.154). In further stratified analyses, *CTLA-4* +49A/G polymorphism was found to be significantly associated with susceptibility to NSCLC in these models (dominant model: OR=1.404, 95%CI: 1.074-1.836; allele model: OR=1.273, 95%CI: 1.034-1.565; homozygous model: OR=1.553, 95%CI: 1.044-2.310; heterozygous model: OR=1.308, 95%CI: 1.062-1.611).

**Conclusion:**

*CTLA-4* +49A/G polymorphism were not associated with the risk of lung cancer but might be a risk factor only in NSCLC.

Lung cancer is one of the malignant tumors that seriously threaten human health and the incidence of lung cancer is increasing year by year worldwide^[1]^. Current studies suggest that complex interactions between genetic anomalies and environmental factors are associated with cancer pathogenesis. It is a multi-factor, multi-stage and multi-gene process^[2, 3]^. Thus, searching for susceptible genes to establish high-risk population and achieve early prevention and treatment is one of the focuses of lung cancer research.

A considerable number of studies have showed that immune system plays a vital role in cancer development and progression^[4]^. Tumor cells can escape the attack of the epidemic system through immunosuppressive checkpoints^[5, 6]^. T lymphocyte and nature killer (NK) cells play a key role in tumor immune surveillance and are regulated by some immune suppressive or stimulus-related molecules^[7]^. Thus, gene mutations associated with cellular immune function and regulating the activation and proliferation of T lymphocytes and NK cells may be involved in cancer susceptibility^[8, 9]^.

Cytotoxic T lymphocyte associated antigen 4 (CTLA-4), also known as CD152, is a member of the immunoglobulin superfamily and an important immune checkpoint gene, which is mainly expressed in Treg and activated T cells^[10]^. The key function of CTLA-4 is to control CD4^+^, CD8^+^ T cells and regulatory T cells (Treg)^[11]^. CTLA-4 has high homology with the co-stimulatory molecule receptor (CD28) on the surface of T cells^[12]^. It shares B7 ligand with CD28. The binding of CTLA-4 and B7 can inhibit the binding of B7 and CD28, interrupt the activation of T cells and participate in the negative regulation of immune response^[13]^. Many studies have shown that CTLA-4 inhibits T cell proliferation, induces activated T cell apoptosis and controls Treg^[14]^. In addition, inhibitors targeting CTLA-4 can block the binding of CTLA-4 to B7, inhibit the production of T cell suppression signals and enhance specific anti-tumor immune response. Therefore, the gene status of CTLA-4 may be related to the occurrence of cancer which is a combination of genetic susceptibility and external factors^[10]^.

There are more than 100 single nucleotide polymorphisms in *CTLA-4* gene, such as + 49 adenine (A)/guanine (G), -318 cytosine (C)/thymine (T), -1611 G/A, -1722 T/C, 10223 G/T polymorphisms, *etc*^[12, 15, 16]^. Among the cancer susceptibility studies, *CTLA-4* +49 A/G polymorphism is the most widely studied mutation point. Some studies have shown that *CTLA-4* +49 A/G polymorphism is associated with the risk of various cancers, such as breast cancer^[17, 18]^ and cervical cancer^[19]^. However, the relationship between *CTLA-4*+49 A/G polymorphism and lung cancer is still unclear. Several studies have drawn contradictory conclusions^[10, 20-26]^. Considering the importance of CTLA-4 in tumorigenesis and the limitations of single study, we conducted a comprehensive meta-analysis of published studies to derive a more precise and objective estimation of the relationship between *CTLA-4* +49A/G polymorphism and the risk lung cancer.

## Materials and Methods

### Identification and eligibility of relevant studies

A systematic literature search was conducted on PubMed, Embase, Wanfang, China National Knowledge Infrastructure (CNKI) and Web of Science databases until June 29, 2019, using the following key words and search strategies: ("CTLA-4" or "cytotoxic T-lymphocyte-associated antigen 4" or "+49 A/G" or "rs231775") and ("polymorphisms" or "mutation" or "variants") and ("lung cancer" or "pulmonary cancer" or "lung neoplasms" or "non-small cell lung cancer (NSCLC)" or "NSCLC"). These terms were arranged into different combinations used for search. Only published studies written in English with available full-text were included in this *meta*-analysis.

The selection criteria were as follows: (1) studies involved the association between *CTLA-4* +49 A/G polymorphism and the risk of lung cancer; (2) studies designed as a case-controlled study; (3) contained available data on the frequency of genotypes including odds ratios (ORs) and 95% confidence intervals (CIs). In addition, the studies that did not meet the inclusion criteria were excluded. Data for the *meta*-analysis were available from 8 articles including 9 case-controlled studies (Fig 1).

**1:**
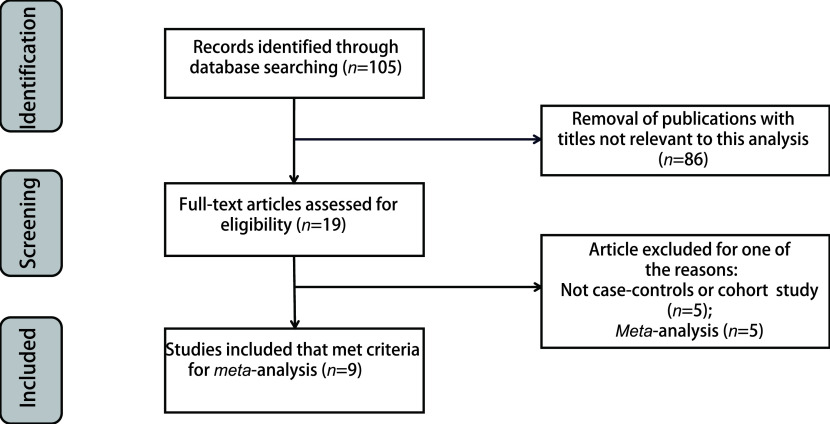
Flow diagram of the study selection process

### Data extraction

Two investigators extracted data from the eligible studies independently according to the inclusion criteria. When confronted with conflict, an agreement was settled by discussion with the third reviewer. For each study, the extracted information included: the first author's surname, year of publication, country of origin, ethnicity, sample size of case and control groups, source of controls, histological types, genotyping methods, genotype distributions and *Hardy-Weinberg equilibrium* (HWE) test. Ethnicities were categorized as Asians or Caucasians.

### Statistical analysis

The pooled ORs and its 95%CIs were calculated to evaluate the strength of association between *CTLA-4* gene polymorphisms and the risk of lung cancer under five genetic models: allele model, homozygous model, heterozygous model, dominant model and recessive model. The pooled ORs were calculated using the fixed-effects model or the random-effects model and a *P* < 0.05 was considered to indicate statistically significant heterogeneity. When the *P* > 0.05, the pooled ORs were calculated using the fixed effects model based on the Mantel-Haenszel method. Otherwise, the random-effects model with the DerSimonian-Laird method was chosen in this meta-analysis. Stratification analyses were performed according to ethnicity (divided into Asians and Caucasians), histological type, source of controls and sample size. In addition, Funnel plots and *Begg's* test were used to evaluate publication bias. All the statistical tests were performed using Stata 11.0 software (Stata Corporation, College Station, TX, USA). A *P* < 0.05 was considered statistical significance.

## Results

### The characteristics of published studies

Following the selected criteria, a total of 105 studies were initially identified through a primary search of PubMed, Embase, Wanfang, CNKI and Web of Science databases and reference lists. Among the studies, 8 full-text articles, including 9 case-control studies harbouring a total of 4, 430 cases with lung cancer and 5, 198 controls met the inclusion criteria and were included in the *meta*-analysis for further evaluation, which were accrued between September 2008 and April 2020. Besides, the distribution of genotypes among the controls was consistent under HWE. The flowchart of literature search and selection procedure is shown in Fig 1. The baseline characteristics of the studies and the distribution of genotype are comprehensively listed in Tab 1 and Tab 2. Among the included studies, 6 were based on Asian populations and 3 on Caucasian populations. Genotyping was performed using polymerase chain reaction - restricted fragment length polymorphisms (PCR-RFLP) in 6 studies, TaqMan in 2 studies and singlenucleotidepolymorphism(SNP) scan kit in 1 study.

**1:** General characteristics of qualified studies included in the *meta*-analysis

First author	Year	Contry	Ethnicity	Number of cases (Male/Female)	Number of controls (Male/Female)	Histological types	Source of controls	Genotyping	Gene polymorphism	HWE
Sun	2008	China	Asian	1, 163 (814/349)	1, 132 (781/351)	NA	Population based	PCR-RFLP	+49A > G, -1722A > G, -1661T > C, -318C > T, +6230G > A	0.909
Sun	2008	China	Asian	1, 032 (770/262)	1, 021 (771/250)	NA	Population based	PCR-RFLP	+49A > G, -1722A > G, -1661T > C, -318C > T, +6231G > A	0.152
Khaghanzadeh	2010	Iran	Caucasians	127 (105/22)	124 (89/35)	Squamous cell carcinoma, adenocarcinoma, small cell lung cancer, carcinoid tumors, non-well differentiated lung cancer	Population based	PCR-RFLP	+49A > G, -1722A > G, -1661T > C, -318C > T, +6232G > A, +1822C > T	> 0.050
Karabon	2011	Poland	Caucasians	208 (142/66)	326 (165/161)	Non-small cell lung cancer	Population based	PCR-RFLP	+49A > G, +319C > T, +6230G > A, +10223G > T	0.880
Antczak	2013	Poland	Caucasians	71 (46/25)	104 (NA/NA)	Non-small cell lung cancer (squamous cell carcinoma, adenocarcinoma, large cell carcinoma)	Population based	TaqMan	+49A > G, -318C > T	> 0.050
Liu	2015	China	Asian	231 (145/86)	250 (142/108)	Non-small cell lung cancer	Hospital based	PCR-RFLP	+49A > G	> 0.050
Ma	2015	China	Asian	528 (352/176)	600 (394/206)	Non-small cell lung cancer	Hospital based	PCR-RFLP	+49A > G, -318C > T	> 0.050
Chen	2017	China	Asian	549 (391/158)	611 (433/178)	Adenocarcinoma, squamous cell carcinoma, small cell lung cancer	Hospital based	TaqMan	+49A > G	> 0.050
Chen	2017	China	Asian	521 (287/234)	1, 030 (588/442)	Non-small cell lung cancer (adenocarcinoma, non-adenocarcinoma)	Hospital based	SNPscan Kit	+49A > G, -1722A > G, +6230G > A	> 0.050
PCR-RFLP: polymerase chain reaction-restriction fragment length polymorphism; HWE: *Hardy-Weinberg* equilibrium.										

**2:** Distribution of *CTLA-4*+49A > G polymorphisms genotype and allele among lung cancer patients and controls

Study	Year	Cases (*n*)						Controls (*n*)				
		GG	AG	AA	G	A		GG	AG	AA	G	A
Sun	2008	509	519	135	1, 537	789		563	488	81	1, 614	650
Sun	2008	468	439	125	1, 375	689		493	438	90	1, 424	618
Khaghanzadeh	2010	13	44	66	70	176		7	47	68	67	183
Karabon	2011	34	106	68	174	242		72	145	107	289	359
Antczak	2013	27	25	19	79	63		22	33	49	77	131
Liu	2015	77	101	53	255	207		51	91	108	193	307
Ma	2015	74	282	172	430	626		72	306	222	450	750
Chen	2017	268	231	50	767	331		279	264	68	822	400
Chen	2017	254	219	47	727	313		504	431	93	1439	617

### Quantitative synthesis results

Overall, the strength of association between *CTLA-4* +49 A/G genetic polymorphism and lung cancer risk was evaluated using the pooled ORs and 95%CIs based on five genetic comparison models. A summary of the *meta*-analysis results for the 9 studied *CTLA-4* polymorphism and lung cancer susceptibility is provided in Tab 3.

**3:** *Meta*-analysis results for the included studies of the association between *CTLA-4*+49 A > G polymorphism and risk of lung cancer

Variables	Study	Dominant model				Recessive model				Allele model				Homozygous model				Heterozygous model		
		OR (95%CI)	*P*	*I*^2^ (%)		OR (95%CI)	*P*	*I*^2^(%)		OR (95%CI)	*P*	*I*^2^ (%)		OR (95%CI)	*P*	*I*^2^(%)		OR (95%CI)	*P*	*I*^2^(%)
Total	9	1.037 (0.925-1.161)	0.536	84.4		0.968 (0.888-1.055)	0.456	75.1		0.992 (0.933-1.054)	0.787	87.1		0.980 (0.857-1.121)	0.767	86.3		1.023 (0.906-1.154)	0.716	75.4
Ethnicity																				
Asians	6	1.070 (0.726-1.577)	0.732	88.7		1.052 (0.864-1.280)	0.614	76.7		1.075 (0.874-1.322)	0.494	89.8		1.096 (0.697-1.724)	0.690	89.2		1.049 (0.753-1.461)	0.778	82.8
Caucasians	3	1.311 (0.815-2.112)	0.265	63.5		1.380 (0.576-3.303)	0.470	80.4		1.245 (0.760-2.040)	0.684	82.8		1.584 (0.602-4.165)	0.351	80.6		1.194 (0.864-1.649)	0.283	14.5
Histological type																				
Non-small cell lung cancer	7	1.404 (1.074-1.836)	0.013	68.1		1.259 (0.967-1.641)	0.088	70.0		1.273 (1.034-1.565)	0.023	81.4		1.553 (1.044-2.310)	0.030	76.5		1.308 (1.062-1.611)	0.011	40.4
Small cell lung cancer	2	0.868 (0.432-1.743)	0.690	45.1		1.074 (0.711-1.623)	0.734	0.0		0.943 (0.619-1.437)	0.786	42.1		1.135 (0.570-2.261)	0.719	0.0		0.849 (0.429-1.680)	0.637	36.8
Source of controls																				
Population -based	5	0.949 (0.648-1.390)	0.788	79.5		0.953 (0.728-1.248)	0.727	69.7		0.990 (0.797-1.229)	0.925	80.3		0.965 (0.590-1.580)	0.889	81.4		0.913 (0.665-1.254)	0.573	66.1
Hospital-based	4	1.392 (0.970-1.997)	0.072	77.5		1.216 (0.959-1.541)	0.106	63.7		1.238 (1.977-1.570)	0.077	84.6		1.490 (0.967-2.297)	0.071	77.7		1.315 (0.968-1.788)	0.080	64.9
Sample size																				
≥500	5	0.906 (0.664-1.235)	0.532	80.4		0.957 (0.827-1.108)	0.558	57.8		0.965 (0.833-1.119)	0.641	79.3		0.900 (0.630-1.285)	0.563	80.9		0.912 (0.697-1.191)	0.498	70.8
< 500	4	1.593 (0.950-2.671)	0.077	79.8		1.507 (0.798-2.844)	0.206	79.6		1.408 (0.896-2.211)	0.138	87.5		1.900 (0.855-4.222)	0.115	83.7		1.464 (0.964-2.224)	0.074	63.5

### CTLA-4 +49 A/G and Lung Cancer Risk

In the present *meta*-analysis, the combined results of all analyses showed that the pooled OR of nine studies was 1.037 (95%CI: 0.925-1.161, *P*=0.536) for the dominant model, 0.968 (95%CI: 0.888-1.055, *P*=0.456) for the recessive model, 0.992 (95%CI: 0.933-1.054, *P*=0.787) for the allele model, 0.980 (95%CI: 0.857-1.121, *P*=0.767) for the homozygote model and 1.023 (95%CI: 0.906-1.154, *P*=0.761) for the heterozygote model, indicating no significant association between *CTLA-4* +49 A/G mutation and lung cancer susceptibility (Fig 2 A-E). Next, subgroup analyses by ethnicity, histological types, source of controls and sample size were performed. In the subgroup analysis, +49 A/G variant exhibited a significant association with an increased risk of NSCLC in these models (dominant model: OR=1.404, 95%CI: 1.074-1.836; allele model: OR=1.273, 95%CI: 1.034-1.565; homozygous model: OR=1.553, 95%CI: 1.044-2.310; heterozygous model: OR=1.308, 95%CI: 1.062-1.611), but no significant results were detected in the Caucasian populations (Tab 3). In addition, when the studies were stratified by ethnicity, source of controls and sample size, no significant differences were found in all genetic models.

**2:**
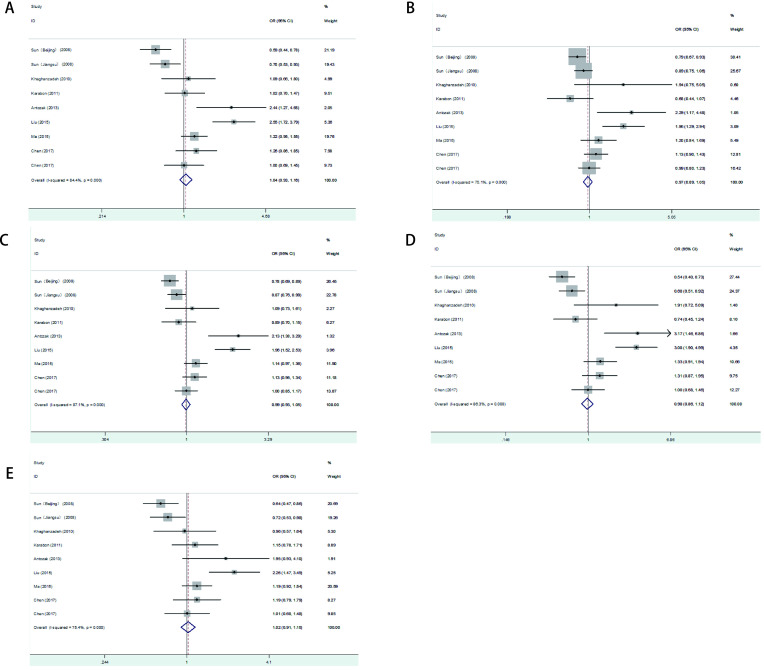
Forest plot of *CTLA-4*+49 A > G polymorphism and lung cancer risk in different genetic comparison models. A: dominant model; B: recessive model; C: allele model; D: homozygous model; E: heterozygous model.

### Test of heterogeneity

For *CTLA-4* +49 A/G, significant heterogeneity was observed after data were pooled (dominant model: *P* for heterogeneity=0.000, *I*^2^=84.4%; Tab 3). In the stratified analyses based on ethnicity, the heterogeneity disappeared in Caucasians group (dominant model: *P* for heterogeneity=0.065, *I*^2^=63.5%; Tab 3). When the subjects were stratified on histological type, the heterogeneity disappeared among with population-based small cell lung cancer (dominant model: *P* for heterogeneity=0.177, *I*^2^=45.1%; Tab 3).

### Publication bias

Funnel plot and *Begg's* test were utilized to evaluate the potential publication biases of the studies involved in the *meta*-analysis. As shown in Fig 3, the shapes of funnel plots showed no evidence of publication bias in the model. Moreover, *Begg's* test provided further statistical evidence for the absence of publication bias, indicating that the results of the *meta*-analysis were reliable.

**3:**
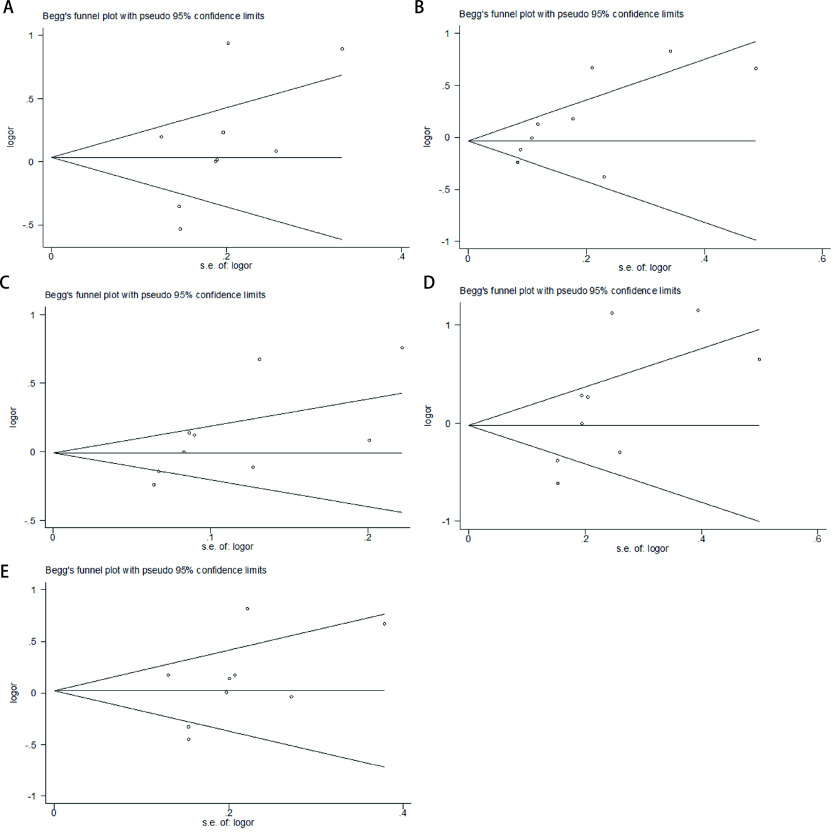
Funnel plot of *CTLA-4*+49 A > G polymorphism and lung cancer risk in dominant model (A), recessive model (B), allele model (C), homozygous model (D), heterozygous model (E).

### Discussion

As one of the important immunoglobulin superfamily genes, *CTLA-4* is always involved in the development and progression of multiple cancers. In recent years, the study of *CTLA-4* polymorphisms involved in the tumorigenesis increased rapidly due to interest in genetic susceptibility to cancer^[10]^. Polymorphism in the *CTLA-4* gene may confer predisposition to cancer. So far, many previous studies have been carried out to investigate the relationship between +49 A/G polymorphism in *CTLA-4* gene and the risk of cancer; however, the data have indicated conflicting results. The *meta*-analysis was performed to evaluate more precise results.

In this *meta*-analysis, a total of 9 eligible and original case-control studies, examined the associations of *CTLA-4* +49 A/G polymorphism and lung cancer risk. Our results indicated that +49 A/G polymorphism did not appear to have a significant association in the overall risk of lung cancer. Because the results of *meta*-analysis may be influenced by ethnicity, histological type, source of controls and sample size, we performed subgroup analyses. The results from stratified analysis indicated that an effect modification of cancer risk was observed in NSCLC by histological types. The associations were not observed in different ethnic population, different source of controls and different sample size.

CTLA-4 acts as a negative regulator of T-cell proliferation and activation through inducing Fas-independent apoptosis of activated T cells, retarding T cells at G_1_ phase in cell cycle and reducing both interleukin (IL)-2 and IL-2 receptor productions^[27, 28]^. Targeting CTLA-4 with a type of monoclonal antibodies in immunotherapy was a prospective therapeutic approach in many types of tumor by enhancing the activation and expansion of antitumor T cell^[29, 30]^. Thus, CTLA-4 may involve in cancer development and progression and exerted an important function in cancer immunosurveillance. The current *meta*-analysis results showed an increased risk of NSCLC for carriers of the A allele. The +49 A/G polymorphism of *CTLA-4* refers to a threonine (Thr) to alanine (Ala) substitution in the leading peptide of CTLA-4 receptor^[31]^. The studies reported that the 49G allele reduced CTLA-4 production than the 49A allele because of its lower messenger ribonucleic acid (RNA) efficiency and this polymorphism enhances the combination between CTLA-4 protein and its ligand B7.1. Individuals with 49GG genotype may lead to greater T-cell proliferation and stronger binding to ligand B7.1 than that with 49 AA genotype^[10]^. According to our current results, these findings revealed that the Thr-to-Ala change in CTLA-4 may be related with the risk of NSCLC.

The heterogeneity and publication bias are of importance which may affect the results of *meta*-analysis. Significant heterogeneity existed in overall comparisons in the dominant model. After subgroup analyses by ethnicity, histological type, source of controls and sample size, the heterogeneity effectively was decreased or almost removed in some subgroups, suggesting different gene-environment factors effect on different histological type or different population. In the *meta*-analysis, publication bias was analyzed by *Begg's* funnel plots and the *Egger's* test and no significant publication bias was detected, suggesting the reliability of our results.

In addition, some limitations should be considered. First, the number of studies was limited, which may affect the power to reveal a reliable association. In the future, large numbers of studies need to be conducted to validate these association. Second, all case-control studies were from Asia and Caucasians, thus our results may be only applicated to these ethnic groups. Third, only published studies were recruited and publication bias might have occurred ineluctably. Fourth, data were not performed to further stratified analysis by other factors, such as environmental and lifestyle factors, because the information extracted from the primary publication was finite.

In conclusion, the results from the *meta*-analysis demonstrated that *CTLA-4* +49 A/G polymorphism was a risk factor for NSCLC. Future more large-scale and well-designed studies with functional evaluations should be carried out to definite the results and investigate the molecular mechanisms of CTLA-4 modify cancer risk.
